# Supervised learning techniques for dairy cattle body weight prediction from 3D digital images

**DOI:** 10.3389/fgene.2022.947176

**Published:** 2023-01-05

**Authors:** Grum Gebreyesus, Viktor Milkevych, Jan Lassen, Goutam Sahana

**Affiliations:** ^1^ Aarhus University, Center for Quantitative Genetics and Genomics, Aarhus, Denmark; ^2^ Viking Genetics, Randers, Denmark

**Keywords:** dairy cow, bodyweight, precision livestock farming (PLF), 3D images, machine learning (ML)

## Abstract

**Introduction:** The use of automation and sensor-based systems in livestock production allows monitoring of individual cows in real-time and provides the possibility of early warning systems to take necessary management actions against possible anomalies. Among the different RT monitoring parameters, body weight (BW) plays an important role in tracking the productivity and health status.

**Methods:** In this study, various supervised learning techniques representing different families of methods in the machine learning space were implemented and compared for performance in the prediction of body weight from 3D image data in dairy cows. A total of 83,011 records of contour data from 3D images and body weight measurements taken from a total of 914 Danish Holstein and Jersey cows from 3 different herds were used for the predictions. Various metrics including Pearson’s correlation coefficient (*r*), the root mean squared error (RMSE), and the mean absolute percentage error (MAPE) were used for robust evaluation of the various supervised techniques and to facilitate comparison with other studies. Prediction was undertaken separately within each breed and subsequently in a combined multi-breed dataset.

**Results and discussion:** Despite differences in predictive performance across the different supervised learning techniques and datasets (breeds), our results indicate reasonable prediction accuracies with mean correlation coefficient (r) as high as 0.94 and MAPE and RMSE as low as 4.0 % and 33.0 (kg), respectively. In comparison to the within-breed analyses (Jersey, Holstein), prediction using the combined multi-breed data set resulted in higher predictive performance in terms of high correlation coefficient and low MAPE. Additional tests showed that the improvement in predictive performance is mainly due to increase in data size from combining data rather than the multi-breed nature of the combined data. Of the different supervised learning techniques implemented, the tree-based group of supervised learning techniques (Catboost, AdaBoost, random forest) resulted in the highest prediction performance in all the metrics used to evaluate technique performance. Reported prediction errors in our study (RMSE and MAPE) are one of the lowest in the literature for prediction of BW using image data in dairy cattle, highlighting the promising predictive value of contour data from 3D images for BW in dairy cows under commercial farm conditions.

## Introduction

Over the past decades, one of the major changes the dairy sector has witnessed has been a reduction in the number of farms with a simultaneous increase in the average herd size (Lowder et al., 2016; Martin and Egon, 2018). With such increases in the average herd size, individual cow management, as well as measurement for traits of interest, is becoming more challenging and labor demanding (Barkema et al., 2015). As a result, the application of automation and sensor systems has increased among dairy farmers as a means to reduce labor costs and improve the management of large herds (Bewley, 2010; Eastwood et al., 2016; Milkevych et al., 2022).

The use of automation and sensor systems in livestock production, commonly termed precision livestock farming (PLF) (Friggens and Thorup, 2015; Berckmans, 2017), allows monitoring of individual cows in real time (RT) and provides the possibility of early-warning systems to take necessary management actions against possible anomalies (Song et al., 2018). Among the different RT-monitoring parameters, body weight (**BW**) plays an important role in tracking the productivity and health status and provides an insight into lactating cows’ energy balance of individual cows (Mäntysaari and Mäntysaari, 2015; Lassen et al., 2018). It is an integral trait determined mostly by an individual’s genetics and feeding conditions and is subject to temporal variability due to several factors such as physiological state (such as lactation), health status, and other environmental stressors (de Vries et al., 1999; Collard et al., 2000; Mäntysaari and Mäntysaari, 2015). Monitoring BW can allow farmers to make management decisions aimed at early interventions regarding cows’ health status (van der Tol and van der Kamp, 2010).

Several studies explored the possibility of predicting BW using animal morphological features acquired with novel approaches including computer-vision techniques (Song et al., 2018; Jang et al., 2020; Weber et al., 2020). Such approaches cover a variety of techniques to generate predictive features based on dairy cows’ morphology. In particular, contour data based on 2-dimensional (2D) vision (Weber et al., 2020), thermal vision (Stajnko et al., 2008), stereo vision using multiple calibrated 2D cameras (Tasdemir et al., 2011), and 3-dimensional (3D) vision using one or multiple 3D cameras have been previously explored (Marinello et al., 2015; Salau et al., 2016; Song et al., 2018; Jang et al., 2020). Investigation on the promise of computer vision for the prediction of BW in cattle remains a dynamic research topic where combined efforts promise better prediction accuracy toward mainstreaming image-based systems for prediction and monitoring BW in dairy cattle. However, a comparison across studies in predictive performance is hampered by the use of different data sizes and structures (time-series (TS), single-record samples, *etc.*), different breeds of cattle, inconsistent use of validation and data-split strategies, as well as metrics to compare model performance (Pearson’s correlation coefficient, *R* ^2^, root mean squared error (RMSE), the mean absolute percentage error (MAPE), the average magnitude of error (MAE), *etc.*).

Additionally, previous studies on the prediction of BW in cattle based on digital image data mostly used general linear regression models (e.g., Tasdemir et al., 2011; Jang et al., 2020). However, the linear regression methods have been shown to have limitations in handling several predictors, as in the case of automated high-throughput phenotyping, with complex and often nonlinear relationships among these predictors (Comrie, 1997). In comparison, machine-learning (ML) techniques are shown to be better in handling big data and modeling several predictors simultaneously addressing the issue of non-linearity among variables (Shahinfar and Kahn, 2018). The ML is a fast evolving research area and numerous supervised and unsupervised learning techniques with potential application to livestock phenomics are proposed. Nonetheless, only a handful of such techniques are applied to the prediction of BW from image data in dairy cattle (Shine and Murphy, 2022). Moreover, the few studies available on prediction of BW in dairy cattle were breed-specific; all of which focused on the Holstein breed (Tasdemir et al., 2011; Kuzuhara et al., 2015; Hansen et al., 2018; Song et al., 2018). To the best of our knowledge, no study investigated predictive ability image data for BW in the Jersey dairy cattle or used a combined Jersey–Holstein dataset.

In this study, by using one of the largest training datasets used for the prediction of BW in the literature (> 80,000 records from two dairy cattle breeds and three different commercial farms), we investigate the performance of various supervised learning techniques in the prediction of dairy cattle BW using contour data from 3D images. We implement various data-filtering and -splitting methods that accommodate time-series data and used various metrics (Pearson’s correlation coefficient; RMSE, MAPE, *etc*.) for a robust evaluation of the prediction ability of the various supervised learning methods used in this study.

## Materials and methods

### Ethics approval statement

All procedures to collect 3D images and body weight data were based on non-invasive methods as part of routine farm management and hence, no specific permission was required.

**FIGURE 1:**
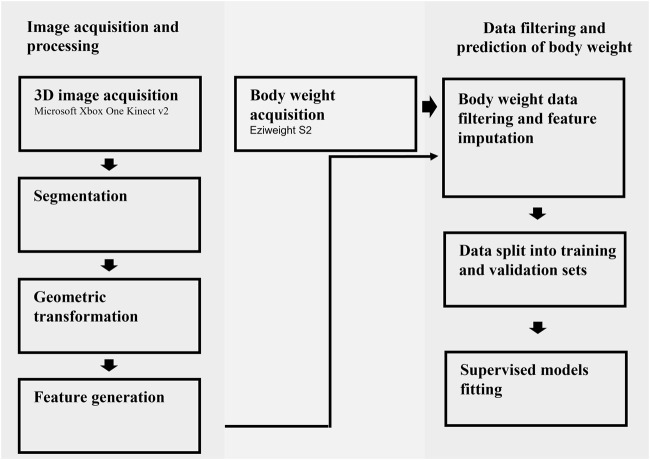
A flowchart of all the steps involved in the image acquisition, processing, and prediction of body weight.

The methods implemented in this study involved eight major steps for image acquisition and processing as well as data filtering and model fitting (Figure 1).

### Sampled cows and body weight data

Data used in this study included a total of 83,011 records of contour data from 3D images and BW measurements taken from a total of 914 Danish Holstein and Jersey lactating cows from three different herds (WO 2020260631A1, 2020). Of the 914 cows used for obtaining image and BW data, 521 cows were from the Danish Holstein and 393 from the Danish Jersey breeds. The data for the Jersey cows were from two different commercial farms while all the Holstein data came from a single separate farm in Denmark. The number of cows and the distribution (range and mean) of the number of records available per cow in each breed and in the combined Holstein–Jersey dataset are presented in Table 1. The age of the cows during the data collection ranged between 22 and 136 months. BW was measured using a weighing scale installed on the route after the milking parlor in such a way that the BW of passing individual cows is always recorded right after being milked.

**TABLE 1:** The number of cows and the distribution (min, mean, and max) of the number of records per cow in each breed and the combined dataset.

Breed	No. of cows	No. of records	No. of records per cow		
			Mean	Min	Max
Jersey	393	31,935	81	1	263
Holstein	521	51,076	98	2	241
Combined	914	83,011	91	1	263

### 3D digital image acquisition and processing

The reference unit consists of a single 3D camera using Time of Flight technology (Microsoft Xbox One Kinect v2) to create a 3D image and a Radio Frequency Identification (RFID) reader (Agrident Sensor ASR550). A DELL T630 128 GB RAM server with a 3090 RTX graphics card was used for the data analysis. These were installed in a narrow corridor with a time-based trigger system that allocates all images taken within 3 s of reading an RFID to the associated ear tag. This system ensured that one reference image was obtained from each cow when they passed through the corridor. The corridor has been narrowed further than a normal exit corridor to avoid anomalies during image acquisition; for instance, two cows exiting together or cows turning around and exiting at oblique angles. The 3D camera was placed at a height of 3.4 m above floor level, directly above the passing cows. At the same position as the camera, a bespoke walking scale (Eziweight S2) was installed to make individual BW recordings of the cow that was passing. The scale was calibrated to 50 and 100 kg using two 50 kg blocks.

Before any cows enter the system, the fixed interior in the image of an empty corridor is annotated. In that way, anything that enters an image will be noticed as a change from the annotated picture and be considered a cow. The first step in the image process is to estimate features from the geometric information in the 3D images, which are useful for separating the individuals. All points within the cow circumference are located in a point cloud, so each pixel in this region of the 3D image is transformed into the corresponding spatial 3D coordinates. The calibration procedure is primarily done to remove distortions due to perspective.

**FIGURE 2:**
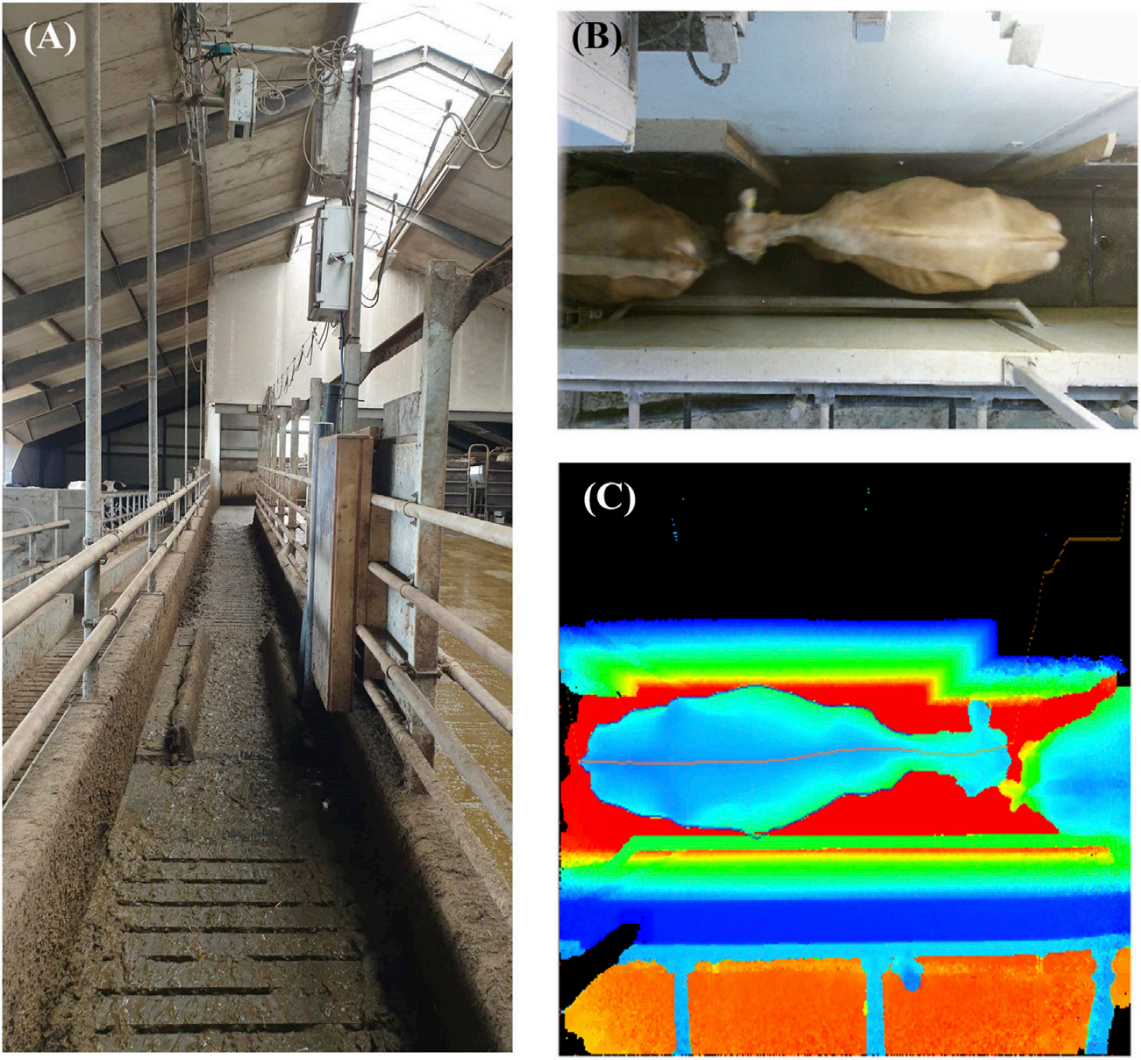
An illustration of the corridor where the 3D images are taken **(A)**, an example cow in the corridor **(B)**, and a heatmap of the highest point on the spine of the example cow **(C)**.

The process starts by finding the circumference and spine of the cow in the raw uncorrected 3D images. The circumference is defined as the last pixel before the image sees the annotated floor. Across the back of the cow, the highest point is found and named the spine. This is simply the highest point across the whole corridor. Figure 2 presents an image of the corridor where 3D images are taken (A), an example cow in the corridor (B), and a heatmap of the highest point on the spine of the example cow (C). The feature generation process starts by finding the points on the corrected depth image lying 3, 5, 10, and 15 cm below the spine level of the cow. So how far left or right respectively should you go from the spine to drop 3, 5, 10 or 15 cm is given. This describes the contour of the back of each cow. Because of the length standardization descripted previously, 100 spots are placed for each of the 3, 5, 10, and 15 cm features. In total, 900 spots for each image. The variables used to predict BW are the distance between 3, 5, 10, and 15 cm, respectively, from left to right across the spine of the cow. The height is measured perpendicular to the spine of the cow to make the features invariant with respect to position and orientation. Cubic smooth splines are fitted to the points corresponding to each distance to reduce noise.

**FIGURE 3:**
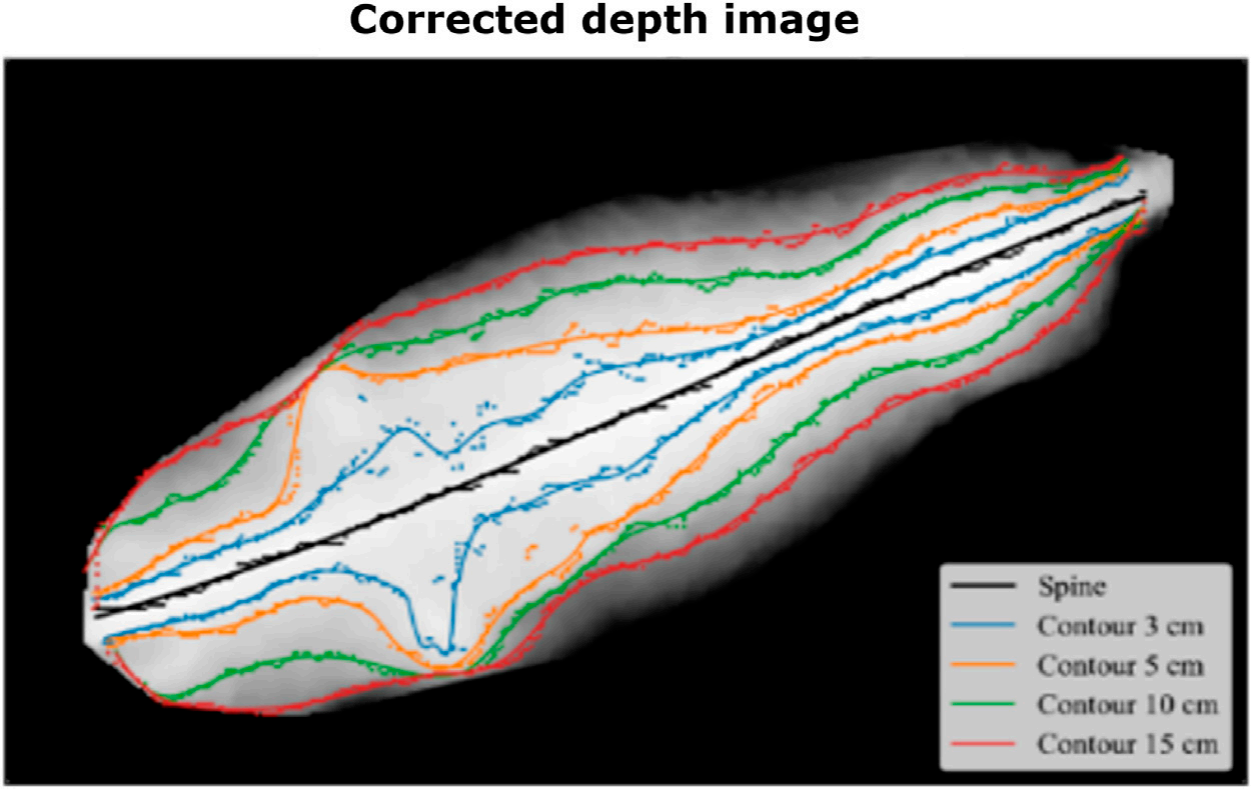
An illustration of the data from an image after correction for the annotated floor. All images are standardized in length and width. The highest point across the image is the spine and these points can be found all the way down the back. Afterward, the distance you have to go left or right respectively to drop 3, 5, 10 and 15 cm is found, and again, 100 points are found and noted. The data used to predict bodyweight are the distance from left to right between the 3, 5, 10 and 15 cm point.

An illustration of some of the acquired images and examples of these contours are presented in Figure 3. The raw spine features are generated by measuring the distance between the intersection of the spine normal and the spines on each side of the cow. The raw spine features are normalized to correct for anatomically differences. All programming was carried out using the Python software.

### Data filtering

Data used in this study include (i) the sets of individual time series (TS) of cows’ BW measurements and (ii) associated sets of contours (resulted from image processing) for each individual’s TS records. The data records include some proportion of unavoidable incorrect records due to on-site measurements (improper position of cows on weighing scale, malfunctions in the weighing scale, image quality, *etc.*). Therefore, implementation of data filtering, considered here as detection and removal and/or imputation of incorrect records, is an important primary step in the automated data processing pipeline.

The methods used in our study for filtering data were, to some extent, based on prior knowledge of BW characteristics in Jersey and Holstein dairy cows such as expected average of BW at a specific age and breed-specific growth rates. That means, the prior information was used in tuning and/or adapting the selected filtering methods to handle TS data of BW records. The records (of cows’ weights) are provided with (i) herd and farm conditions; (ii) animals’ ages; and (iii) breed-specific characteristics of growth dynamics.

Three different filtering approaches were used and tested for the best performance (filtering quality) in the study. The first approach was based on the modified Z-score (**ZS**) method (Iglewicz and Hoaglin, 1993).

The time-series body weight data for a specific cow is the sequence of records sampled continuously within a specified time interval $\Delta_{T}$ : $D_{j}=\left\{\left(t_{i},w_{i}\right)\;|\;t_{i}\in \left[t_{1},t_{n}\right],\;t_{n}=t_{1}+n\Delta_{T};\;\Delta_{T}=const;\;i=1,\ldots ,n\right\}$ , where $t_{i}$ is the time the body weight $w_{i}$ is measured, n is the number of records, and j=1, …, m where m is the number of cows studied. Note that, the data $D_{j}$ are not necessary to be complete in the sense of all $\left(t_{i},w_{i}\right)$ pairs exist, these are the missing records.

Assume that $W∼N\left(\mu_{w},\sigma_{w}^{2}\right)$ , where $W\subset D_{j}$ is the body weight subset of $D_{j}$ , $\mu_{w}$ is the mean of W , and $\sigma_{w}^{2}$ is the variance of W ; then $Z∼N\left(0,1\right)$ , where $Z=\left(W-\mu \right)/\sigma$ . The Z-scores of the records $\left\{w_{i}\right\}$ can be defined as (Iglewicz and Hoaglin, 1993)

$$z_{i}=\left(w_{i}-\mu^{*}\right)/s,$$

where $\mu^{*}$ is the sample median, s is the estimator defined as the median of the absolute deviations about the sample median normalized by the constant

$$s=\frac{median_{i}\left\{\left|w_{i}-\mu^{*}\right|\right\}}{0.6745}.$$

The normalization constant 0.6745 is required because the expectation $E\left(median_{i}\left\{\left|w_{i}-\mu^{*}\right|\right\}\right)$ equals 0.6745σ for a large n (Iglewicz & Hoaglin, 1993).

Finally, the records are labeled outliers when the following condition is satisfied:

$$\left|z_{i}\right|>\tau_{ZS},$$

where $\tau_{ZS}$ is a predefined threshold value which is evaluated in relation to the prior information, such as breed- and age-specific median weight of a particular TS. In this study, the threshold range was $\tau_{ZS}=2.0-3.5$ .

The second-implemented data-filtering approach (**CL1**) was based on the mean shift clustering (MSC) algorithm (Comaniciu and Meer, 2002). Here, the appropriate use of MSC in the case of $D_{j}$ is based on the assumption that the subset $W_{r}\in W$ of the records (where subscript r indicates records are not outliers) forms a unique cluster while other records (the outliers) form one or more clusters separated from the $W_{r}$ -cluster.

As in the case of the ZS method, we use only the W subset of $D_{j}$ (the BW records for a specific cow without time stamps). Such one-dimensional data are further subjected to the MSC algorithm. Here, we refer to Comaniciu and Meer (2002) for all the mathematical details of the algorithm. In our study, we use the *sklearn.cluster* python module for the practical implementation of the MSC algorithm.

Finally, the resulting cluster is not labeled outlier when the following condition is satisfied:

$$\tau_{CL1}-\sigma_{CL1}\leq \tilde{w_{c}}\leq \tau_{CL1}+\sigma_{CL1},$$

where $\tilde{w_{c}}$ is the cluster specific median weight, $\tau_{CL1}$ is the threshold based on breed- and age-specific weight expectations (in this case, within-breed and age mean weights from the data), $\sigma_{CL1}$ is the standard deviation of the threshold.

The third data-filtering approach was based on clustering using the dynamic changes of animals’ weights, such as expected breed-, age-, and farm-specific daily weight gain (**CL2**).

Let us suppose that the initial record $D_{j0}=\left\{\left(t_{l},w_{l}\right)\in D_{j},\;l=1,\;..,k;k\ll n\right\}$ , where k is the number of selected initial records, consists of both correctly measured values and outliers. Furthermore, we assume that the outliers in $D_{j}$ (as the results of measuring errors) are determined by the same factors.

Data clustering is realized by iterative calculation of the dynamical BW change for every pair $\left(t_{i},w_{i}\right)$ in $D_{j}$ using $\left(t_{l},w_{l}\right)\in D_{j0}$ as the starting (reference) values; and compare the calculated estimates to the expected dynamical changes. Hence, the clustering is based on whether the records are lying within the expected temporal changes of BW gain/loss (specific for a known breed, age, and herd conditions) or not. Initially, the procedure creates two cluster sets which are subjected to the same iterative procedure aimed at finding possible embedded clusters (checking if smaller clusters exist).

The following estimator is used to estimate the dynamical change of BW in $D_{j}$ :

$${d_{w}}_{i}=\frac{w_{i+1}-w_{0}}{t_{i+1}-t_{0}}\;\text{for}\;i=0,\ldots ,n-1,$$

where $w_{i+1}$ and $t_{i+1}$ are the elements of $D_{j}$ ; $w_{0}$ and $t_{0}$ are the elements of $D_{j0}$ . Note that the estimates ${\left\{{d_{w}}_{i}\right\}}_{l}$ are calculated k times using k pairs of $D_{j0}$ as fixed $w_{0}$ and $t_{0}$ values.

The resulting record is labeled outlier (inherited by the outlier cluster) when the following condition is satisfied:

$$\left|{d_{w}}_{i}\right|>\tau_{CL2},$$

where $\tau_{CL2}$ is the breed- and age-specific threshold of the temporal changes based on prior information. Here, the threshold-expected daily gain/loss value of 1 kg is used since the average daily weight changes, due to net energy intake, are not expected to exceed 1 kg (Jensen et al., 2015).

**FIGURE 4:**
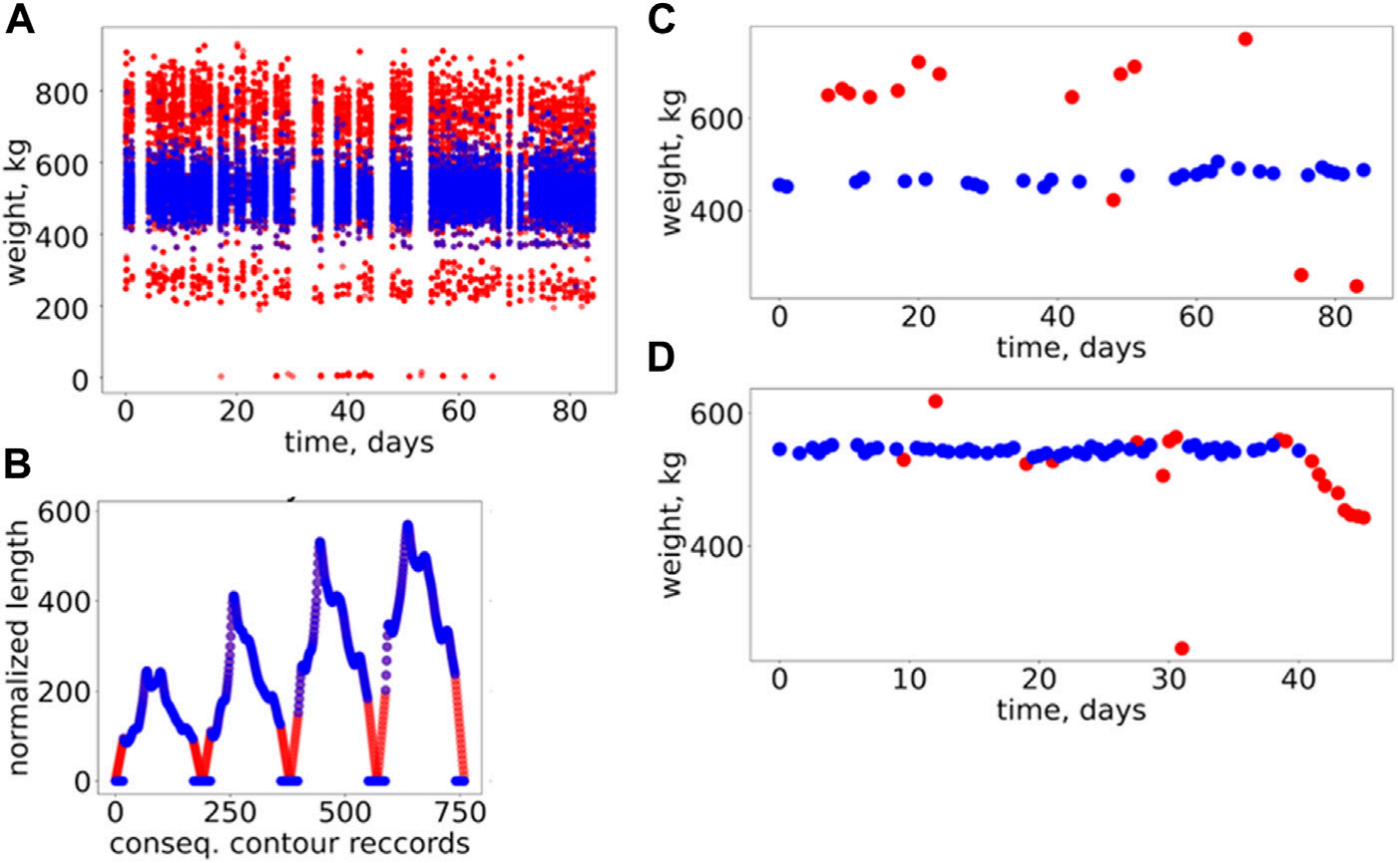
General overview of the results of data-filtering and imputation. **(A)** Changes of body weight over time for the combined datasets (all data depicted in the plot) for Jersey and Holstein; here the original (raw) data is represented by red dots; the same data but without outliers are represented by the blue dots; the outliers were filtered out using the CL2 method. **(B)** An example of imputation of contour data for a specific (randomly selected) cow; here, the blue dots indicate the original contour data resulting from the image processing; the red dots indicate an imputed data for the missing records in the original contour data; the plot depicts the complete dataset (for the selected cow) which will be used for modeling; the different colors shown in the plot are just to highlight the imputed part of the contour data. **(C)**, **(D)** Two examples of body weight changes over time for the randomly selected cows; here, the original (depicted by the red dots) data and the data without outliers (the blue dots) are shown together.

The general overview of data filtering is represented in Figure 4 (and more details in Supplementary File S1). In order to determine the best performing data-filtering method, all three methods were implemented and analyzed with respect to the quality of outlier filtering. In this regard, three different simulation pipelines using three filtered datasets (generated according to the specific filtering method) were established. The pipelines involved training and validation of the range of selected models (see all details further in this section). The prediction results (BW for each dataset) were subjected to the quality assessment. Here, the assessment was based on Pearson’s correlations coefficients between the predicted and observed BW. Accordingly, results on the predictive performances of the different models presented in this study are based on the best-performing data-filtering technique (CL2) based on this preliminary assessment. A fully functional Python code for implementing the data-filtering techniques is presented in Supplementary File S2.

In contrast to the BW records, the contour data have limited prior knowledge: all contours are expected to be closed, which means that the first and last records of a normalized distance (width) should approach zero. We used this information for contours’ records filtering and imputation (recover missing records). Because no additional information was available regarding the shape of the contours’ curves, we used simple linear approximation to detect outliers and complete (impute) the missing/incorrect contours’ records (Figure 4B).

### Training and validation strategies

Predictive ability of contour data from 3D images for BW in dairy cows was first investigated separately within each breed and subsequently in the combined multi-breed (Holstein–Jersey) dataset. Within each dataset (Jersey, Holstein, and combined), splitting the data into training and test sets was the first step in the implementation of the prediction models. Accordingly, we implemented approaches including the traditional time-series approach (TS), a modified version of the time-series approach (TS2), and an approach randomly splitting data (RA) for splitting the data into training and test sets. The first two scenarios were based on time-series data splitting such that observations from the training set occurred before their corresponding test set. These were implemented in a two-step manner, such that, the first 80% of cows were randomly selected and records for each of these cows were sorted according to timestamps, while the remaining 20% cows were held out as the test set. Subsequently, for these randomly selected cows, 80% of the records with the earliest timestamps were selected for training. The remaining 20% records from these randomly selected cows were either discarded (**TS**) or added to the test set (**TS2**). Therefore, the main difference between scenario 1 (TS) and scenario 2 (TS2) was whether later records of cows in the training set were included in the test set. In addition, a third data-split scenario was implemented by randomly selecting 80% of individuals for training and excluding all their records from the test set, while the remaining 20% were used as the test set (**RA**).

In the traditional time-series prediction, usually a part at the end of each series is reserved and not used during model generation for later use in model evaluation. This is often called out-of-sample evaluation (Tashman, 2000). Whereas, in machine-learning studies, cross-validation is the most widely used tool in the evaluation of regression and classification methods; studies have demonstrated practical problems and violations of important assumptions such as stationarity in time-series data (Bergmeir and Benítez, 2012). Therefore, here in this study, we apply, in addition to the RA and TS approaches, a modified TS approach in a two-stage manner where we first take a random sub-sample of the data (80%) at a time and apply time-series approaches to split the data into training and test sets. That way, we were able to undertake repeated evaluations (replicates) and still use a part at the end of each series for validation. Except for the predictions using the linear regression techniques (LR, RR, and, to some extent, LA), the modified time-series approach (TS2) for splitting data into training and test sets resulted in higher correlation between predicted and observed BW and low RMSE and MAPE values across the datasets.

### Supervised learning techniques

Several methods chosen to represent different families of techniques in the supervised learning space were compared for predictive performance of BW using contour data.

### Linear regression

Here, we used the ordinary linear regression (**LR**) and the regularization techniques of ridge regression (**RR**) (Hoerl and Kennard, 1970) and the Least Absolute Shrinkage and Selection Operator regression (**LASSO**) (Tibshirani, 1996). The linear regression method is one of the most widely deployed tools for predicting a quantitative response (James et al., 2013), and is often used as a benchmark method in comparing the performance of other ML methods. The RR and LASSO are a family of the linear regression technique with regularization of parameters in the linear regression fit.

### Tree-based regression

We have chosen the random forest (**RF**) (Ho, 1995) and decision tree (**DT**) regressors as well as boosting approaches as representatives of the tree-based family of supervised learning techniques. The tree-based methods are a group of supervised learning techniques that is getting increasing popularity in computational biology (Geurts et al., 2009) and represents the most frequent-used family of techniques in the ML applications to the livestock space (Shine and Murphy, 2022). Regression trees have previously been used for BW prediction in different livestock species including cattle (e.g. Topal et al., 2010). While regression trees are relatively simple for implementation and interpretation, prediction accuracies have often been lower than other supervised learning techniques (James et al., 2013). In contrast, methods such as random forests and boosting use trees as building blocks to construct more powerful prediction models by aggregating many decision trees. Therefore, in addition to the random forest model, we implement two boosting approaches (Adaptive Boosting (AB) and Catboost (CB)) in this study. Catboost (Ostroumova et al., 2018) is developed for an unbiased boosting with categorical features. However, studies have implemented and demonstrated how well-suited CatBoost is for regression problems involving time-series data (Kolesnikov et al., 2019; Hancock and Khoshgoftaar, 2020). For the RF and DT regressors, the sklearn *ccp_alpha* parameter for Minimal Cost-Complexity Pruning was optimized through cross-validation.

### Support vector regression

Support vector **(SV)** machines (Vapnik and Lerner, 1963) have been extensively used in machine learning, primarily for classification, and are being increasingly applied for regression as well (Smola and Schölkopf, 2004).

All implemented supervised learning techniques, except the *Catboost* regressor, were imported from their corresponding packages available in the scikit learn website (https://scikit-learn.org). The Catboost regressor was imported from the open-source *Catboost* package (Ostroumova et al., 2018).

### Evaluation of predictive performance

Predictive performances of the different models were quantified by using Pearson’s correlation coefficient ( r ), root mean squared error (RMSE), and the mean absolute percentage error (MAPE) computed as

$$r=\frac{n\left(\sum_{i=1}^{n}y_{i}{\hat{y}}_{i}\right)-\left(\sum_{i=1}^{n}y_{i}\right)\left(\sum_{i=1}^{n}{\hat{y}}_{i}\right)}{\sqrt{n\left(\sum_{i=1}^{n}y_{i}^{2}\right)-{\left(\sum_{i=1}^{n}y_{i}\right)}^{2}\;}\sqrt{n\left(\sum_{i=1}^{n}{\hat{y}}_{i}^{2}\right)-{\left(\sum_{i=1}^{n}{\hat{y}}_{i}\right)}^{2}\;}},$$ (1)

$$RMSE=\sqrt{\frac{\sum_{i=1}^{n}\left(y_{i}-{\hat{y}}_{i}\right)}{n}},$$ (2)

$$MAPE=\frac{1}{n}\;\overset{n}{\underset{1=1}{\sum }}\left|\frac{y_{i}-{\hat{y}}_{i}}{y_{i}}\right|x\;100\%,$$ (3)

where $y_{i}$ is the reference BW, ${\hat{y}}_{i}$ is the predicted weight from the models, i denotes the $i^{th}$ record, while n is the total record available.

The prediction was undertaken in ten replicates for each dataset (Jersey, Holstein, and combined) and data-split scenario (TS2, TS, and RA), and the average r , RMSE, and MAPE values were reported.

## Results

### Descriptive statistics

**FIGURE 5:**
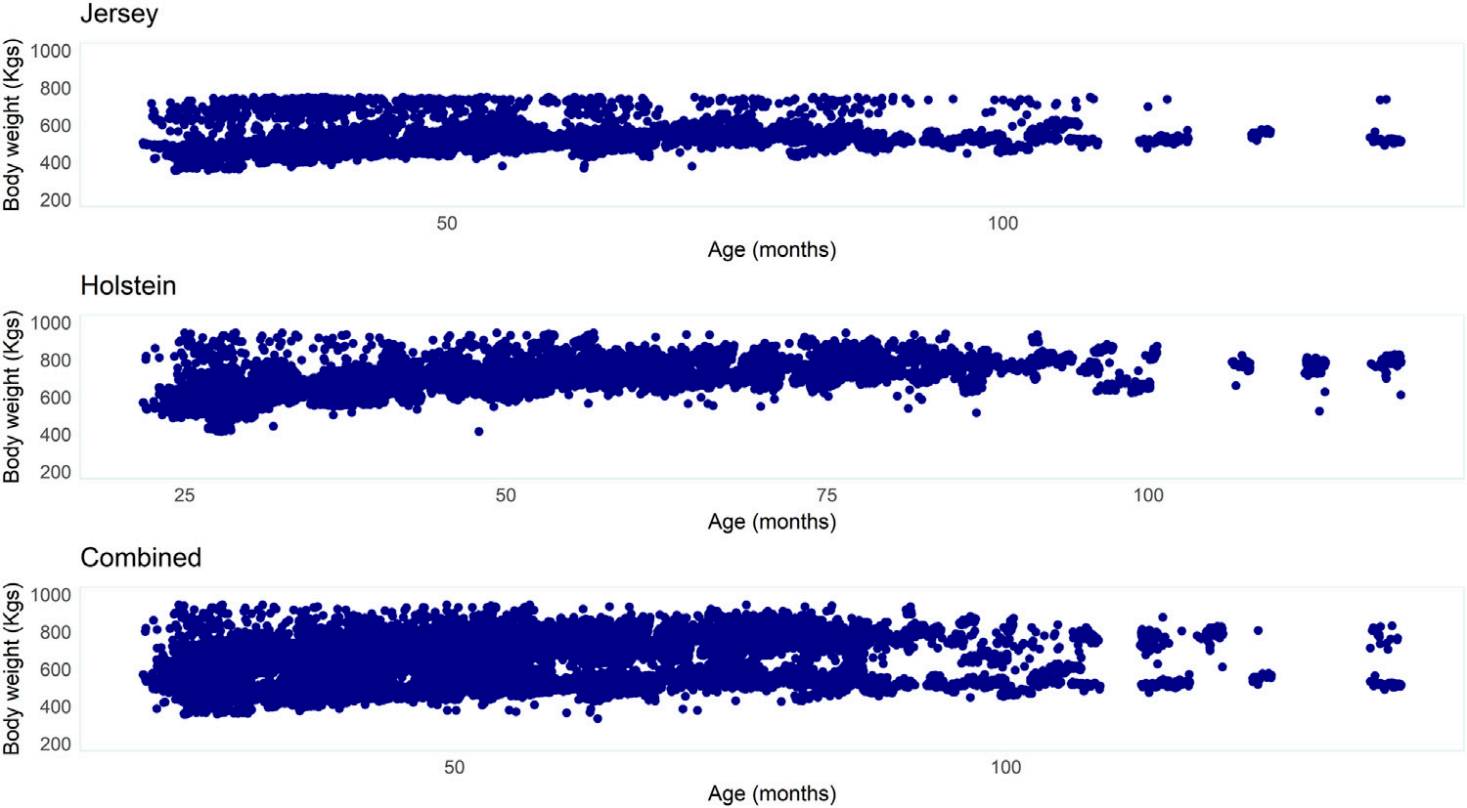
Bodyweight (kg) across ages (months) of cows from the three datasets (Jersey, Holstein, and combined breed).

Descriptive statistics of BW measured in dairy cows according to the breed and the combined dataset are presented in Table 2. The Jersey cows had a mean BW of 558.4 kg, while the Holstein cows had a mean BW of 690.3 kg. The coefficient of variability (CV) observed in BW measurements was comparable in the two breeds while a relatively higher CV was observed in the combined dataset. Figure 5 presents BW across ages of cows in the two breeds and the combined dataset.

**TABLE 2:** Descriptive statistics of the BW measured in Jersey and Holstein cows, as well as the combined dataset.

Farm/breed	Body weight (BW)			
	Min (kgs)	Max (kgs)	Mean (kgs)	CV (%)
Jersey	354	748	558.4	14.4
Holstein	414	942	690.3	12.3
Combined	354	942	646.7	17.4

CV, coefficient of variation.

### Predictive performance of supervised learning techniques

**FIGURE 6:**
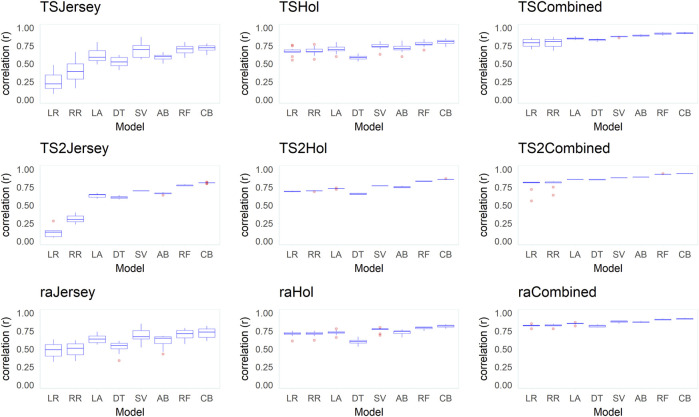
Box plots of correlations (r) between the observed and predicted body weight (BW) from predictions in 10 replicates across the different datasets (Jersey, Holstein, and combined), data-split scenarios (TS2, TS, and RA), and various supervised learning techniques (LR, linear regression; RR, ridge regression; LA, LASSO; DT, decision tree; SV, support vector machine; AB, Adaboost; RF, random forest; and CB, Catboost).

**FIGURE 7:**
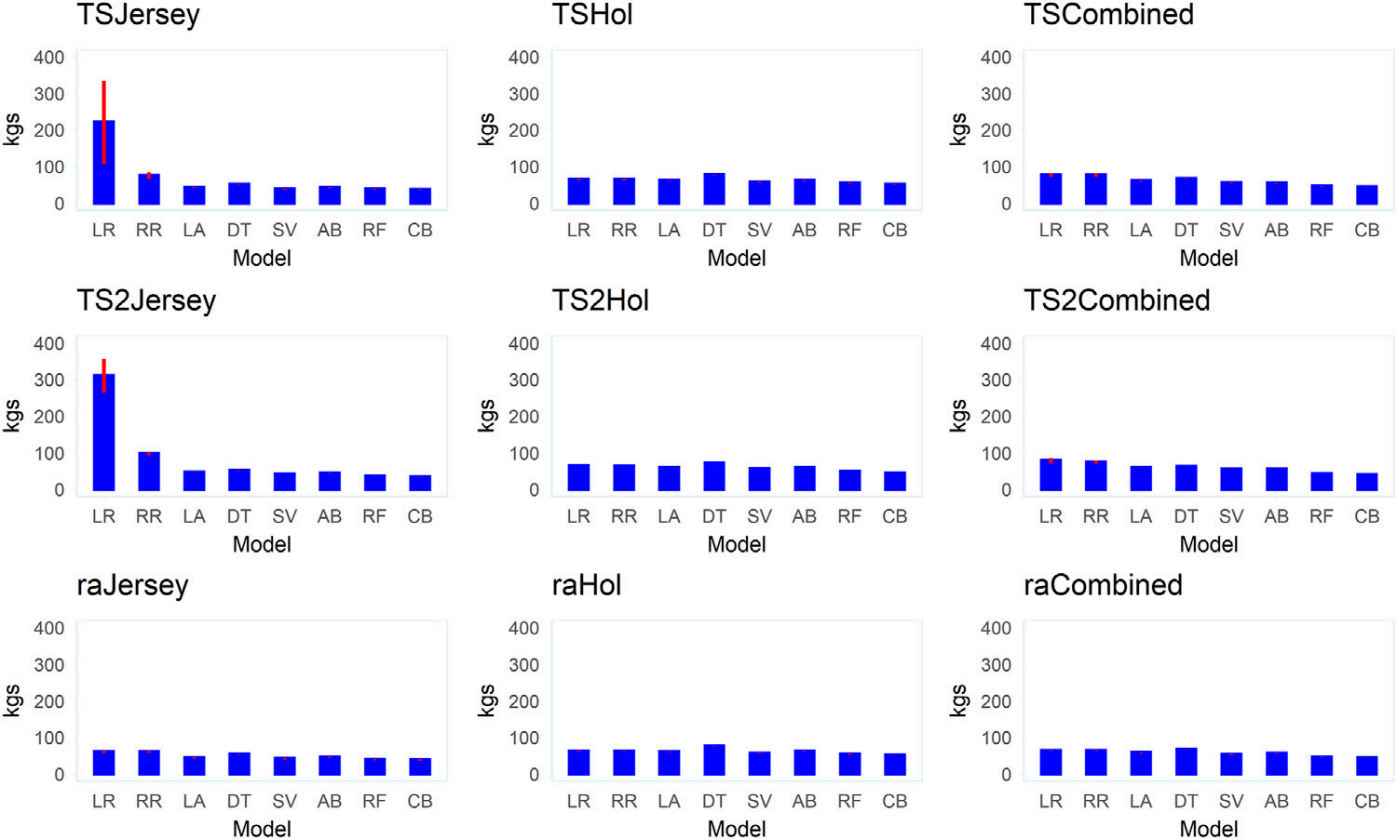
Bar plots of the mean root mean squared error (RMSE) values (+ standard error bars) for the different supervised learning techniques (LR, linear regression; RR, ridge regression; LA, LASSO; DT, decision tree; SV, support vector machine; AB, Adaboost; RF, random forest; and CB, Catboost) across the different datasets (Jersey, Holstein, and combined) and data-split scenarios (TS2, TS and RA).

**FIGURE 8:**
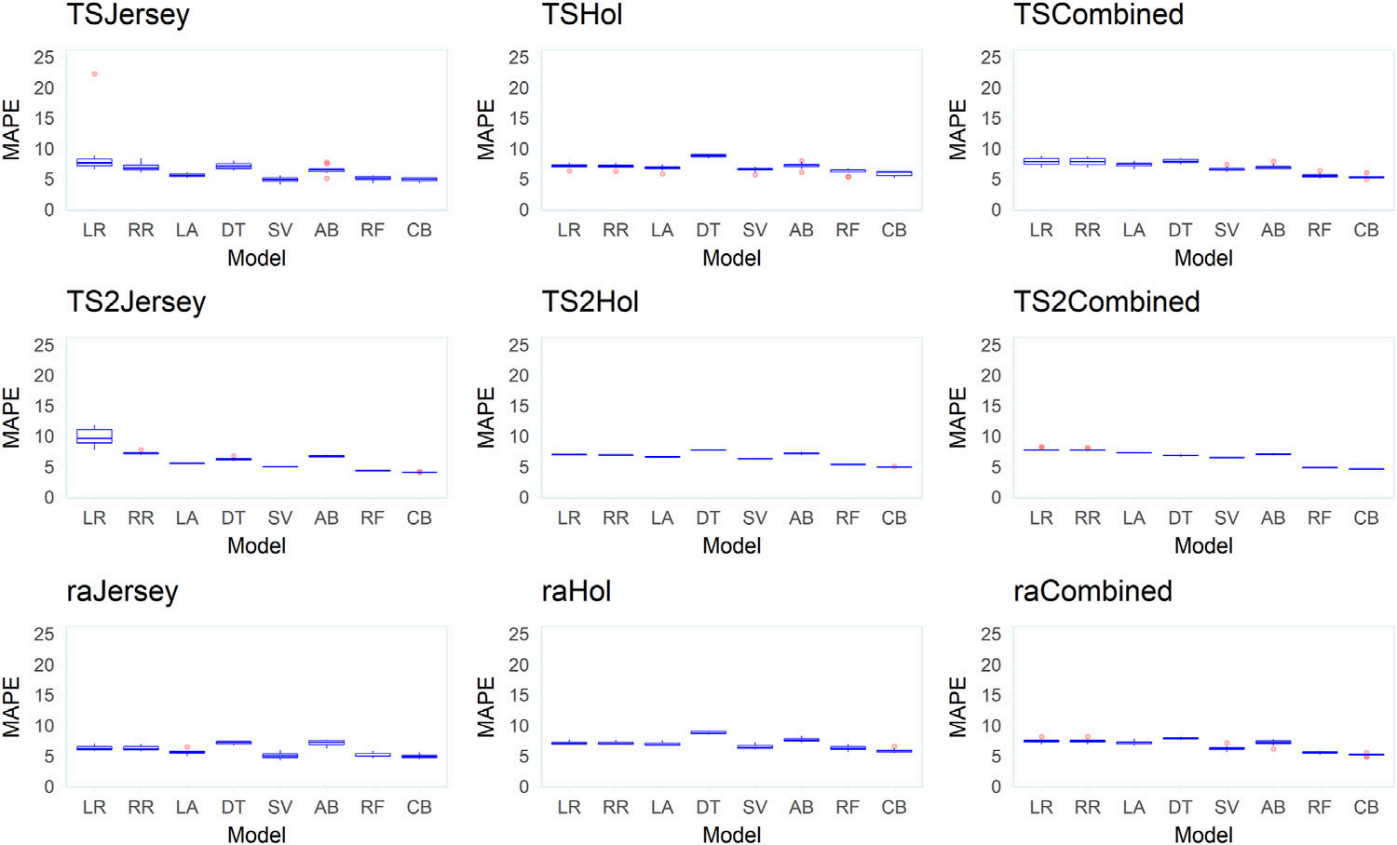
Box plots of the mean absolute percentage error (MAPE) between observed and predicted body weight (BW) from predictions in 10 replicates across the different datasets (Jersey, Holstein, and combined), data-split scenarios (TS2, TS, and RA), and various supervised learning techniques (LR, linear regression; RR, ridge regression; LA, LASSO; DT, decision tree; SV, support vector machine; AB, Adaboost; RF, random forest; and CB, Catboost).

Figures 6–8 present mean correlation coefficients ( r ) between predicted and observed BW, RMSE, and MAPE values, , from 10 replicates across the different datasets, data-split scenarios (TS, TS2, and RA), and various supervised learning techniques. In general, while the prediction performance varied depending on the dataset (Jersey, Holstein, and combined breeds), learning techniques, data-split scenario, and reasonable prediction accuracies were achieved with a mean correlation coefficient ( r ) as high as 0.94 and MAPE and RMSE as low as 4.0% and 33.0 (kg), respectively. Supplementary File S3 presents Scatter plots of predictions vs. real live weights in the validation sets across the learning techniques and data-splitting methods from one of the ten replicates in the run using the combined data.

Across all datasets and data-split scenarios, the tree-based group of supervised learning techniques (Catboost, AdaBoost, and random forest) resulted in the highest prediction performance in all the metrics used to evaluate the technique performance, followed by the support vector regression technique. The tree-based group of supervised learning techniques also resulted in the lowest standard deviation between the 10 replicates. Of the tree-based methods, Catboost resulted in the highest performance in all the metrics used, while the decision tree regressor resulted in the lowest prediction performance. In most instances (dataset and -split scenario), the support vector method outperformed the decision tree regressor. In the majority of the data-splitting scenarios and datasets, the linear regression methods (classical linear regression, ridge regression, and LASSO) resulted in the lowest prediction performance compared to the rest of families of supervised learning techniques. Within this family, classical linear regression showed the lowest performance in the majority of the scenarios (dataset and data-split method). The exception was in the Holstein dataset, where the decision tree regressor resulted in the lowest predictive performance. Prediction using the linear regression technique was worst in the dataset with the smallest number of records (Jersey) in terms of low Pearson’s correlation coefficient ( = 0.12–0.48) and high RMSE (60–309.8 kg) and MAPE values (6.2–9.8%) across different data-splitting scenarios.

In general, the predictive performance of supervised learning techniques varied according to the datasets. Overall, the highest discrepancy between the learning techniques in predictive performance was observed within the dataset with a relatively smaller number of records (Jersey) where the linear regression group of techniques showed poor performance while the tree-based models were shown to be highly predictive in terms of all metrics used to evaluate models. In the combined (Jersey–Holstein) dataset, where the largest number of records was used in training the supervised learning techniques, and differences in predictive performance between the various supervised learning techniques were modest. Predictive performance was the highest in the combined dataset compared to the single-breed Jersey and Holstein datasets across all implemented models. To test if the improvement in predictive ability in the combined dataset was due to the increase in dataset or due to the multi-breed nature of the combined dataset, we implemented an additional test where multi-breed data of similar sizes as the single-breed Jersey and Holstein datasets were used for prediction using 10 replicates. The results showed that prediction using multi-breed data of equal sizes to the single-breed datasets led to slightly lower prediction accuracies in terms of correlation between the predicted and observed BW. However, the differences were neither statistically nor significantly different except in the comparison in the Jersey dataset (combined multi-breed data with equal size as the Jersey breed dataset) using the LR models.

Predictive performance of the implemented supervised learning techniques also varied according to the data-split scenario used to create training and validation subsets (Supplementary Files S4–S6). In general, higher Pearson’s correlation coefficients and lower MAPE and RMSE values were obtained when supervised learning techniques were trained using the training subset created with the TS2 method, followed by TS in comparison to the approach of splitting training and testing subsets randomly (RA).

## Discussion

### Relative performance of different supervised learning techniques

Generally, our results indicate marked differences in the predictive abilities of various supervised learning techniques depending on the dataset (Jersey, Holstein, and combined data) and the data-splitting techniques followed. Relatively higher predictive performance was shown for the tree-based family of supervised learning techniques compared to a wide variety of other techniques implemented in this study to represent different families of methods in the ML space. Boosting methods such as Catboost as well as the random forest performed reasonably well in this study. A similar observation of the outstanding performance of the tree-based techniques in the prediction of cattle BW using various features including 3D image data is previously reported (e.g. Weber et al., 2020). The linear regression group of techniques (LR, RR, and LASSO) generally performed poorly in our study. In general, the relative performance and suitability of different supervised learning techniques may vary, among others, depending on the nature of the data and the relationship between predictors and between predictors and the outcome. The linear regression group of learning techniques tends to perform poorly when there is a nonlinear relationship between the predictors and the outcome (James et al., 2013). CatBoost is a gradient-boosted decision-tree implementation for supervised ML allowing ordered target statistics and ordered boosting (Hancock and Khoshgoftaar, 2020). Gradient boosting is a powerful supervised learning technique that remained an important method for learning problems with heterogeneous features, noisy data, and complex dependencies (Ostroumova et al., 2018).

Repeated records of BW from cows ranging between 22 and 136 months of age were used in training the supervised learning methods in our study. Moreover, the on-access setup of weighing scales along the path to milking parlors might cause some noise in the target variable (BW) in such a way that at times, cows might have placed only the front or rear legs at the time of weight reading and, in some cases, reading might belong to that of two cows both placing some of their legs on the floor attached to the weighing scale. All these combined can introduce heterogeneity and noise in the data. Different approaches have been followed to filter noise and enforce rigorous quality control before implementing the learning techniques. While such approaches can help detect and remove major outliers, it is not possible to eliminate all noise in the data. The differences in the predictive performance of the various learning techniques implemented in this study might largely be due to such unavoidable noises in the data used for training.

### Data-splitting methods and validation metrics matter

Our results also showed differences in the predictive performance of the learning techniques according to the implemented data-split scenario. Except for the predictions using the linear regression techniques (LR, RR, and, to some extent, LA), the modified time-series approach (TS2) for splitting data into training and test sets resulted in higher correlation between predicted and observed BW and low RMSE and MAPE values across the datasets. While both the traditional and modified time-series data-splitting techniques accounted for the time-series nature of the data such that observations from the training set occur before their corresponding test set, they differed regarding the use of a part at the end of each series for validation. In the traditional time-series prediction, usually a part at the end of each series is reserved and not used during model generation for later use in model evaluation. This is often called out-of-sample evaluation (Tashman, 2000); whereas in machine-learning studies, cross-validation is the most widely used tool in the evaluation of regression and classification methods, studies have demonstrated practical problems and violations of important assumptions such as stationarity in time-series data (Bergmeir and Benítez, 2012). The modified-TS approach (TS2) implemented in this study allowed repeated evaluations while still using a part at the end of each series as it was applied in a two-stage manner where we first take a random sub-sample of the data (80%) at a time and apply time-series approaches to split the data into training and test sets. That way, we were able to undertake repeated evaluations (replicates) and still use a part at the end of each series for validation, and thus allowing for better predictive performance.

The comparison across the studies investigating predictive abilities of various supervised learning techniques for the prediction of cattle BW using image data is hampered by the use of different data sizes and structures (time-series, single-record samples, *etc.*), different validation and data-split strategies as well as the use of different metrics to compare model performances (r, RMSE, MAPE, *etc.*).

In our study, the combined use of three metrics (r, RMSE, and MAPE) to evaluate the different supervised learning techniques was chosen to ensure comparability with other studies and for a robust model evaluation as each has different advantages and limitations. In computational biology, it is common to use Pearson’s correlation coefficient (r) as a model selection criterion (González-Recio et al., 2014). However, a limitation is that Pearson’s correlation does not address the bias of predictions (González-Recio et al., 2014). The RMSE is the most popular measure of prediction error which has been used in several studies on the prediction of cattle BW using image data (e.g., Song et al., 2018; Jang et al., 2020; Weber et al., 2020). RMSE is however scale-dependent and hence, a comparison of results between variables or species is not possible. The mean absolute percentage error (MAPE) is, on the other hand, scale-independent and easy to interpret, making it one of the most popular measures of prediction accuracy (Byrne, 2012; Kim & Kim, 2016). In our study, the highest Pearson’s correlation between predicted and observed BW was observed in the combined Holstein–Jersey dataset. This is expected due to the large increase in training data from combining the datasets from the two breeds compared to the within-breed predictions. However, the lowest RMSE value was observed in the Jersey dataset where the smallest training data was used compared to the Holstein or combined datasets. This is due to the fact that Jersey cows are smaller in size when compared to Holstein cows as reflected in the mean BW from the two datasets and the scale-dependent nature of RMSE as an evaluation metric. This suggests the need for caution in the use of scale-dependent metrics such as RMSE for comparison of prediction performance even between different breeds of cattle of the same livestock species.

### Combining datasets is advantageous

Generally, studies on the predictive ability of image data for BW in dairy cattle are scarce, and the few available studies have solely focused on the Holstein breed (Tasdemir et al., 2011; Kuzuhara et al., 2015; Hansen et al., 2018; Song et al., 2018). To the best of our knowledge, no study investigated the predictive ability of image data for dairy cattle BW using multi-breed data. Moreover, most studies relied on numerically small data (number of cows and records). For instance, the image data from 30 or a lower number of cows were used to predict the BW in Holstein cows in the studies of Song et al. (2018) and Kuzuhara et al. (2015). The study of Kuzuhara et al. (2015) used RMSE as the metric to evaluate predictive performance in a linear regression model and report an RMSE value of 42.65 kg while Song et al. (2018) reported the lowest RMSE of 41.2 kg and MAPE of 5.2%. Here in this study, we used a large dataset (> 80K records) across different breeds (Holstein, Jersey, and combined multi-breed data) from different commercial farms to investigate the predictive ability of 3D image data for BW in dairy cattle. We report one of the lowest RMSE (33 kg) and MAPE (4%) values in the literature available on the prediction of dairy cattle BW using contour data from 3D images, indicating that combining data (from different breeds or farms) to increase the size of learning data can improve prediction accuracies.

Often, combining multi-breed data for the prediction of phenotypes is challenging due to differences in the breeds. In this study, we show that the increase in data size due to combining different breeds might offset the disadvantage from the multi-breed nature of the combined dataset given that the combined data is substantially larger than the single-breed data. Several research groups have undertaken studies aimed at predicting BW in dairy cattle using morphometric measurements based on different systems, including 3D images. Across-population combined analysis using available data in the different research groups might substantially improve data size to train learning techniques with substantially reduced prediction errors. Publicly available repositories and databases that store images and corresponding biometrics have previously been suggested (Wang et al., 2021).

### Practical implications and the way forward

In general, our study indicates the promising predictive value of contour data from 3D images for BW in dairy cows under commercial farm conditions. Depending on the supervised learning technique and the datasets used, we show reasonable predictive performances in terms of correlation between predicted and observed BW (r), RMSE, and MAPE, promising an automated and high-throughput prediction of BW in dairy cattle herds.

Use of image data for automated detection of anomalies in cows’ health status or monitoring other short-term changes in cows’ BW requires high sensitivity. While our study reports one of the lowest prediction errors (RMSE as low as 33 kg) in the literature on prediction of BW using image data for dairy cattle, these values might still be considered as high in light of early warning and monitoring systems. However, these RMSE values are in line with the expected fluctuation in body weight within a day for Holstein and Jersey cattle. For instance, dry-matter intake was reported to be approximately between 14 and 20 kg for Danish Holstein cows and between 11 and 16.7 kg for Danish Jersey cows (Li et al., 2016). In addition, the maximum average daily milk yield of 35 and 24 kg/day were reported for Danish Holstein and Jersey cows, respectively (Halachmi et al., 2011). A Holstein cow requires 4–5 kg of water per kg of milk yield (www.lely.com). Therefore, given all these factors introducing massive fluctuations of body weight within a day, the RMSE values reported in this study are within the expected daily weight fluctuations for Holstein and Jersey cows.

Of all potential reasons for higher prediction errors, erroneous records might be difficult to eliminate completely and thus cost prediction accuracies. In this study, we preliminarily assessed three different data-filtering techniques for prediction accuracy and presented results based on data-filtered using the high-ranking method (CL2). Despite a relatively better performance of this outlier detection technique compared to the other two implemented, some irregularities still persist in the filtered data. We used time-series data where some individuals have hundreds of records in different time periods whereas others have only one or few records. High variability was observed in the BW measurement in cows (records) measured at an early age compared to later ages (as shown in Figure 5). Efficient outlier detection and missing-value-interpolation techniques need to be developed to rigorously filter such time-series data. Furthermore, as previously suggested by Nir et al. (2018), considering additional features, based on the image or other data sources, and introducing deep artificial neural network algorithms might allow further improvement in the prediction accuracy of various livestock phenotypes using computer-vision.

## Conclusion

Using a multi-breed dataset consisting of over 80,000 records of contour features from 3D images and BW data, our study reaffirms the promise of image data for rapid and high throughput prediction of BW in dairy cattle. The study implemented and compared the predictive performance of various supervised learning techniques using different metrics and in both within-breed and combined-breed datasets. Our results indicate that combining datasets from different breeds and farms allowed improved prediction accuracy. Our study also show that the tree-based learning techniques, including *Catboost* and random forest led to higher predictive performance, in terms of higher Pearson’s correlation coefficients between predicted and observed BW as well as lower RMSE and MAPE values. Reported prediction errors in our study (RMSE and MAPE) are one of the lowest in the literature for prediction of BW using image data in dairy cattle, highlighting the promising predictive value of contour data from 3D images for BW in dairy cows under commercial farm conditions. Further increasing training data size and development of efficient and tailored outlier detection techniques could allow further reduction of prediction error.

## Data Availability

The raw data supporting the conclusion of this article will be made available by the authors, without undue reservation.
