# Supplementary material for: Supervised learning techniques for dairy cattle body weight prediction from 3D digital images
Source: Front Genet. 2023 Jan 5;13:947176. doi: 10.3389/fgene.2022.947176 (PMC9849234; doi:10.3389/fgene.2022.947176)
Supplement: Supplementary file 1 [file DataSheet2.PDF]

Algorithms for data filtering

Algorithm 1: Modified Z-score

```
# data is the array (map) of data sample
# icol is the column index with data record of interest (weight, for example)
# threshold is the threshold for outlier; it is the number of STDs above which data is considered as an outlier

newarr = {}
for i in id_list:
    arr = data[i]

    md = np.median(arr[:,icol])
    mad = np.median([np.abs(y - md) for y in arr[:,icol]])

    if mad == 0.0:
        newarr[i] = arr
        continue

    z0 = [ np.abs( 0.6745 * (y - md) / mad ) for y in arr[:,icol]]

    z1 = np.empty( shape=(len(z0),1),dtype=np.float64 )

    z1[:,0] = z0[:]

    # remove data above the threshold
    arr = arr[np.all(z1<threshold, axis=1),:]

    newarr[i] = arr

return newarr
```

Algorithm 1: The mean shift clustering

```
# data is the array (map) of data sample
# t_col is the column index with time stamp record
# w_col is the column index with weight record
# w_var is the threshold for outlier; it is the number of STDs above which data is considered as an outlier
# w_mean - determines the cpecific method:
#       1 - AgglomerativeClustering
#       2 - Birch
#       3 - KMeans
#       4 - MiniBatchKMeans
#       5 - MeanShift
#       6 - GaussianMixture

newarr = {}

for i in id_list:
```

```
arr = data[i]
tmp1 = np.empty([arr.shape[0], 2])
tmp1[:,0] = 0
tmp1[:,1] = arr[:,w_col]

if w_mean == 1:
   model = AgglomerativeClustering(n_clusters=3)
   yhat = model.fit_predict(tmp1)
elif w_mean == 2:
   model = Birch(threshold=0.01, n_clusters=3)
   model.fit( tmp1 )
   yhat = model.predict( tmp1 )
elif w_mean == 3:
   model = KMeans(n_clusters=3)
   model.fit( tmp1 )
   yhat = model.predict( tmp1 )
elif w_mean == 4:
   model = MiniBatchKMeans(n_clusters=3)
   model.fit( tmp1 )
   yhat = model.predict( tmp1 )
elif w_mean == 5:
   model = MeanShift()
   yhat = model.fit_predict(tmp1)
elif w_mean == 6:
   model = GaussianMixture(n_components=3)
   model.fit( tmp1 )
   yhat = model.predict( tmp1 )
else:
   print("Wrong method parameter in __cluster2_s(). EXIT.")
   raise SystemExit

# get unique clusters
clusters = unique(yhat)

# create a list of arrays for each particular cluster
# in order to apply Modified Z-score method:
clusterArr = []
for clst in clusters:
   row_ix = where(yhat == clst)
   arr0 = arr[ row_ix, : ][0]
   # ---------- Modified Z-score step ------------------------
   md = np.median(arr0[:,w_col])
   mad = np.median([np.abs(y - md) for y in arr0[:,w_col]])
   if mad == 0.0:
      uTime, indices = np.unique(arr0[:,0], return_index=True)
      clusterArr.append( arr0[indices,:] )
   else:
      z0 = [ np.abs( 0.6745 * (y - md) / mad ) for y in arr0[:,w_col]]
      z1 = np.empty( shape=(len(z0),1),dtype=np.float64 )
      z1[:,0] = z0[:]
```

```
        arr0 = arr0[np.all(z1<w_var, axis=1),:]
        uTime, indices = np.unique(arr0[:,0], return_index=True)
        clusterArr.append( arr0[indices,:] )
    # -----------------------------------
  # Select a proper cluster which follows a breed/herd specific characteristic
  for i2 in range(0,len(clusterArr)):
    apply_the_selection_criteria
    if sutisfied:
      iCluster = i2

  newarr[i] = clusterArr[ iCluster ]

return newarr
```

Algorithm 1: Clustering based on accounting for the dynamic changes in animals' weights

```
# data is the array (map) of data sample
# t_col is the column index with time stamp record
# w_col is the column index with weight record
# w_var is the assumed daily variability of weight
# w_mean - population mean (bread specific), is used as criteria to select a right set among separated (clustered) data sets

newarr = {}
for i in id_list:
  arr_high = []
  arr_low = []
  arr = data[i]
  w_ref = arr[0,w_col]   # reference weight, (kg)
  t_ref = arr[0,t_col]      # reference time, timestamp (seconds)
  tind = 0
  low_weight = 0.0
  high_weight = 0.0

  # Separate data on two distinct sets
  # using a daily weight change as a clustering criteria
  for j in arr[:,w_col]:
    der_w = 0.0
    delta_t = np.abs( float(arr[tind,t_col] - t_ref)/86400 ) # days
    delta_w = np.abs(j - w_ref)

    if delta_t != 0.0:
      der_w = delta_w/delta_t

    if der_w < w_var:
      low_weight = low_weight + j
      arr_low.append(tind) # we are collecting indexes
    else:
      high_weight = high_weight + j
      arr_high.append(tind) # we are collecting indexes
```

```
        tind = tind + 1

    # Here we have to choose one right set
    # among the two separated sets;
    # As a criteria we use either:
    # (i) power of clustered sets, so we select the one which has more data
    # or (ii) we use population mean (w_mean parameter) as a criteria, so
    # the data set whose mean is closest to the population mean is selected
    if w_mean == 0:
        # Here we make choice based on the size of the separated sets
        if len( arr_high ) > len( arr_low ):
            z = np.empty( shape=(len(arr_high),arr.shape[1]),dtype=np.float64 )
            iz = 0
            for jj in arr_high:
                z[iz,:] = arr[jj,:]
                iz = iz + 1
            newarr[i] = z
        else:
            z = np.empty( shape=(len(arr_low),arr.shape[1]),dtype=np.float64 )
            iz = 0
            for jj in arr_low:
                z[iz,:] = arr[jj,:]
                iz = iz + 1
            newarr[i] = z
    else:
        # Here we use the population mean
        if len( arr_high ) == 0:
            h_mean = 0.0
        else:
            h_mean = high_weight/len( arr_high )

        if len( arr_low ) == 0:
            l_mean = 0.0
        else:
            l_mean = low_weight/len( arr_low )

        h = np.abs( h_mean - w_mean )
        l = np.abs( l_mean - w_mean )

        if l > h:
            z = np.empty( shape=(len(arr_high),arr.shape[1]),dtype=np.float64 )
            iz = 0
            for jj in arr_high:
                z[iz,:] = arr[jj,:]
                iz = iz + 1
            newarr[i] = z
        else:
            z = np.empty( shape=(len(arr_low),arr.shape[1]),dtype=np.float64 )
            iz = 0
```

```
            for jj in arr_low:
                z[iz,:] = arr[jj,:]
                iz = iz + 1
            newarr[i] = z

    return newarr
```
