# Supplementary figures and images for: Supervised learning techniques for dairy cattle body weight prediction from 3D digital images
Source: Front Genet. 2023 Jan 5;13:947176. doi: 10.3389/fgene.2022.947176 (PMC9849234; doi:10.3389/fgene.2022.947176)

Jersey

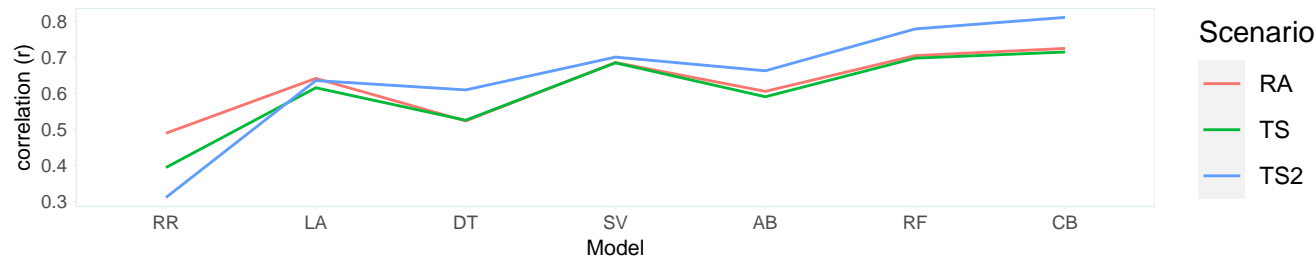

Hol

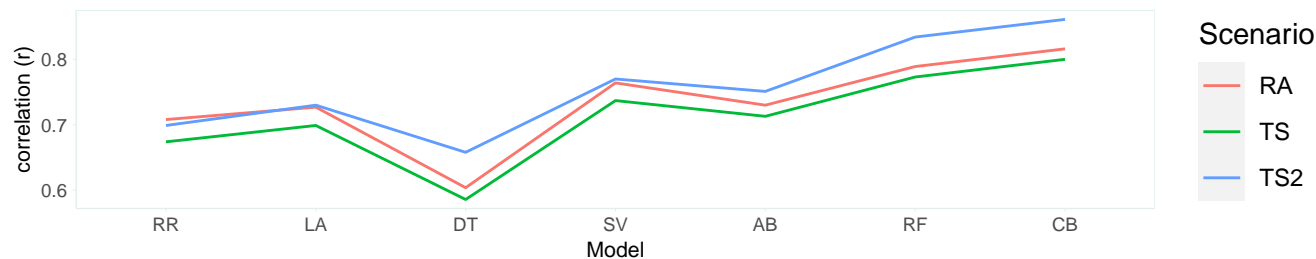

Combined

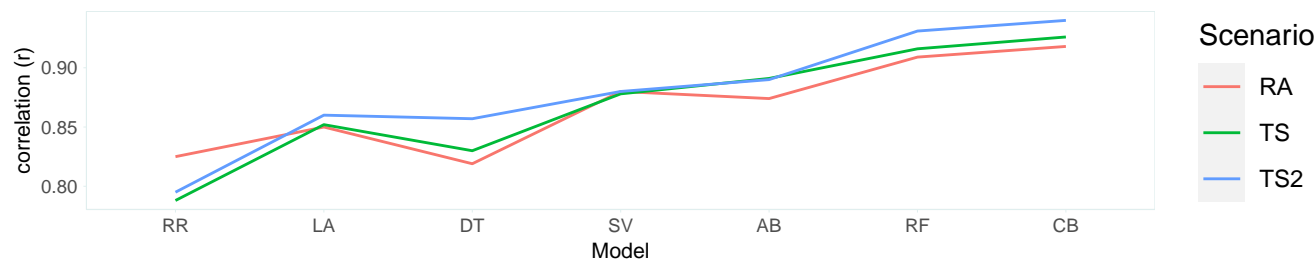

Supplement: Supplementary file 2 [file DataSheet4.PDF]

Jersey

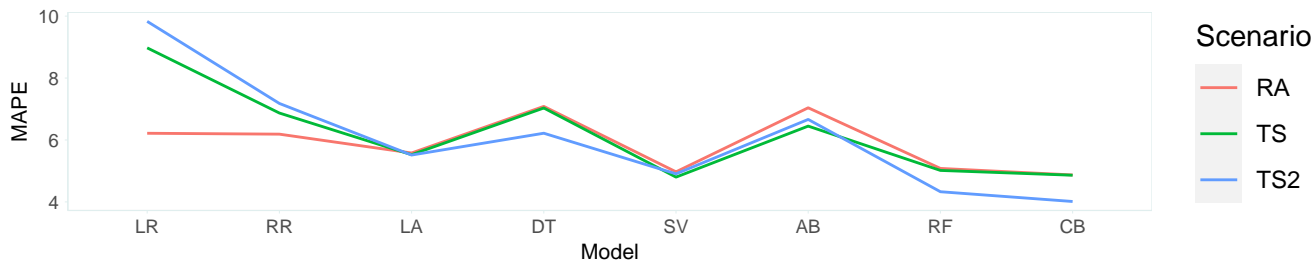

Hol

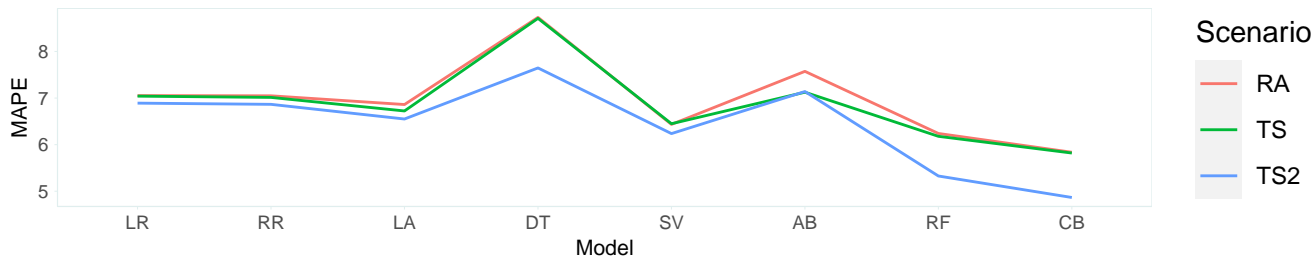

Combined

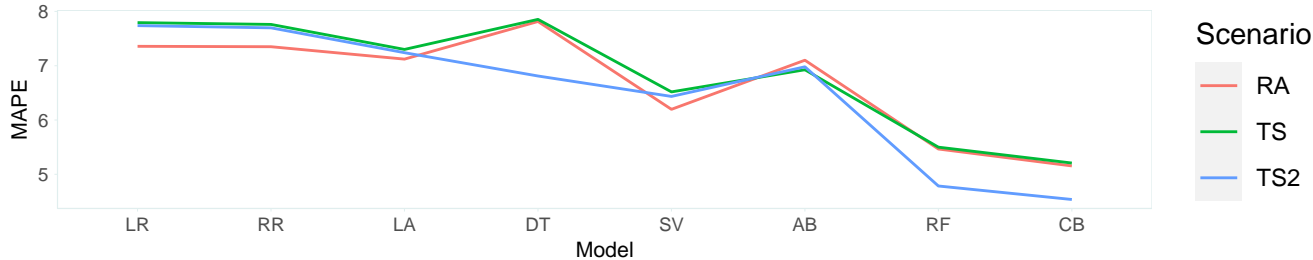

Supplement: Supplementary file 3 [file DataSheet6.PDF]

Jersey

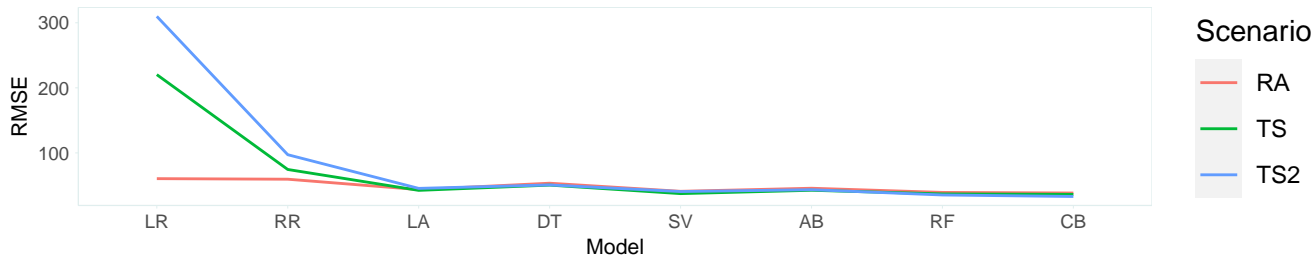

Hol

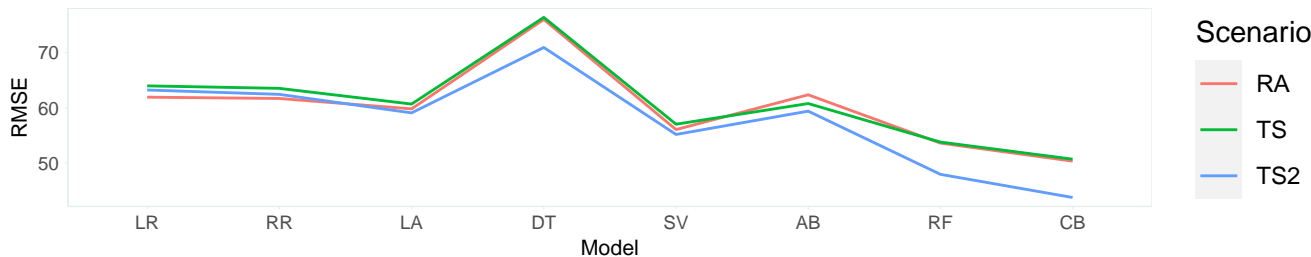

Combined

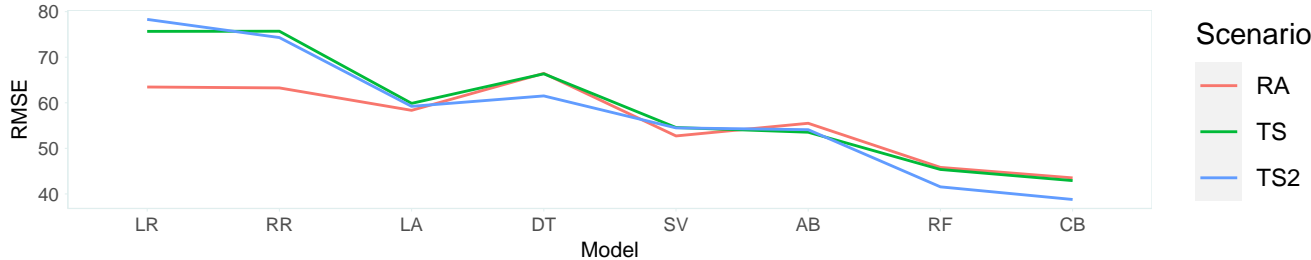

Supplement: Supplementary file 6 [file DataSheet5.PDF]
