# Supplementary material for: Supervised learning techniques for dairy cattle body weight prediction from 3D digital images
Source: Front Genet. 2023 Jan 5;13:947176. doi: 10.3389/fgene.2022.947176 (PMC9849234; doi:10.3389/fgene.2022.947176)
Supplement: Supplementary file 4 [file DataSheet3.PDF]

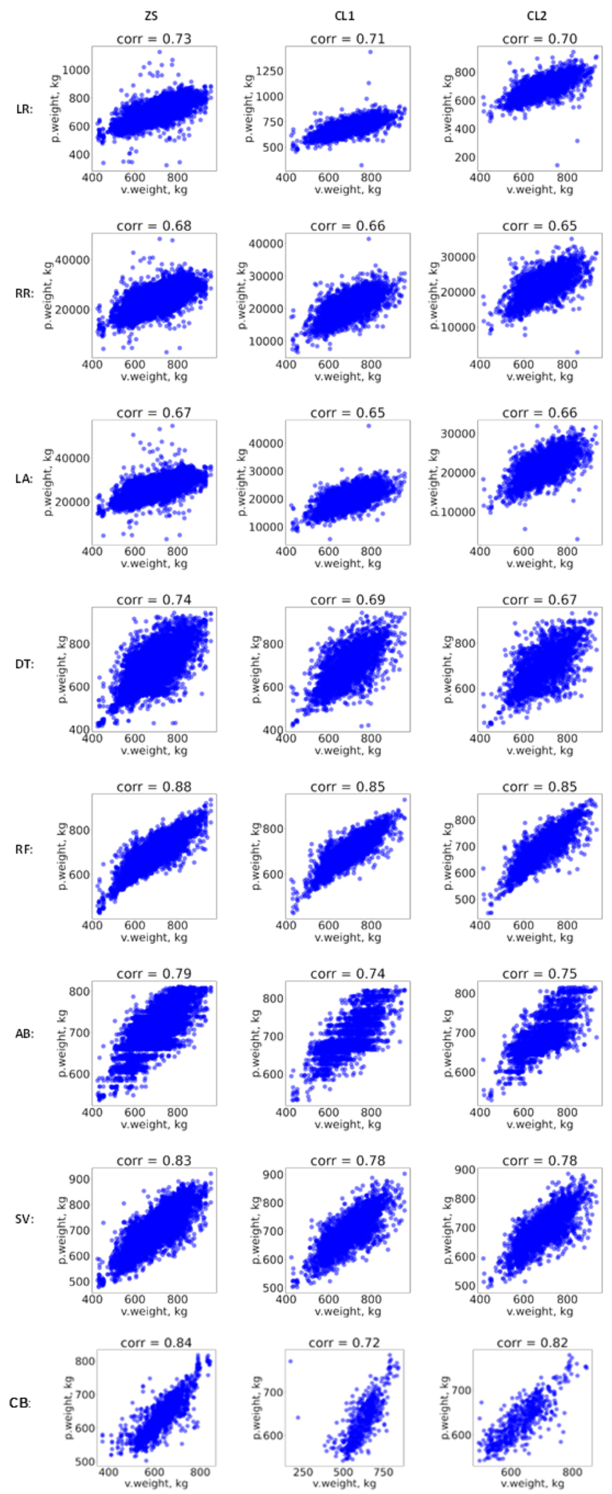

Supplementary File 3. Scatter plots of predictions vs. real live weights in the validation sets across the learning techniques and data-splitting methods from one of the ten replicates in the run using the combined-data. zs – modified Z-score; cl and cl2 – variants of clustering (see the main text for details); LR - linear regression, RR - ridge regression, LA - lasso regression, DT - decission tree regressor, RF - random forest regressor, AB - ada boost regressor, SV - support vector regressor; CB – CatBoost; the numbers above each plot show correlation between predicted weights (p.weights) and validated weights (v.weights) based on one replicate.
