# Supplementary material for: Supervised learning techniques for dairy cattle body weight prediction from 3D digital images
Source: Front Genet. 2023 Jan 5;13:947176. doi: 10.3389/fgene.2022.947176 (PMC9849234; doi:10.3389/fgene.2022.947176)

Supplementary File 1

Fig. S1.1. The general overview of raw and filtered data. Rows represent different data sources; columns represent different filtering (outlier removal) methods: zs – modified Z-score; cl and cl2 – variants of clustering (see the main text for details); the numbers above each plot show amount of outliers detected; red dots – outliers, blue dots – filtered data.

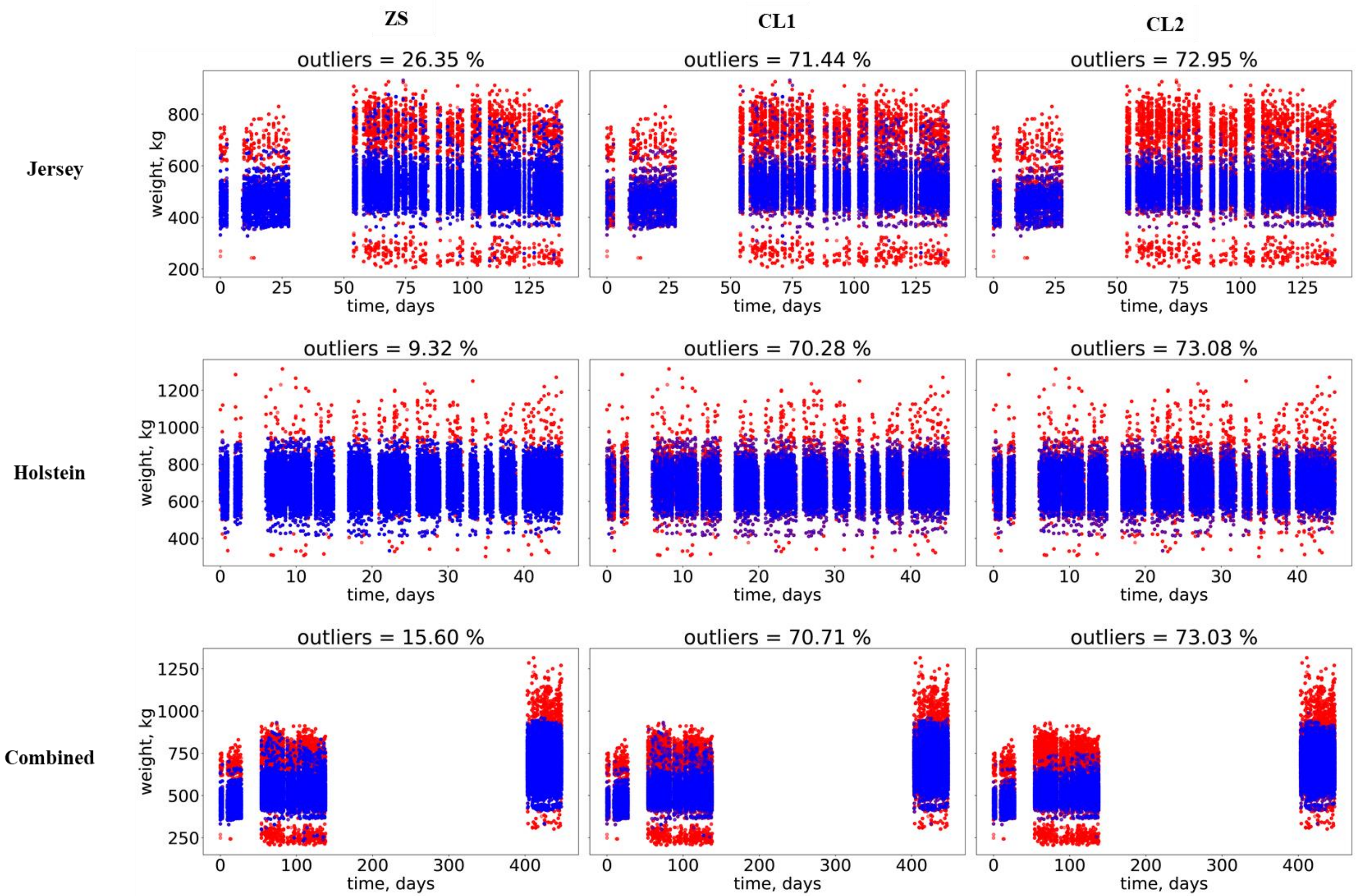

Fig. S1.2. An overview of efficiency/effect of different filtering methods for specific (randomly selected) individuals. Rows represent a specific cow from the relevant data source; columns represent different filtering (outlier removal) methods: ZS – modified Z-score; CL1 and CL2 variants of clustering (see the main text for details); the numbers above each plot show amount of outliers detected; red dots – outliers, blue dots – filtered data.

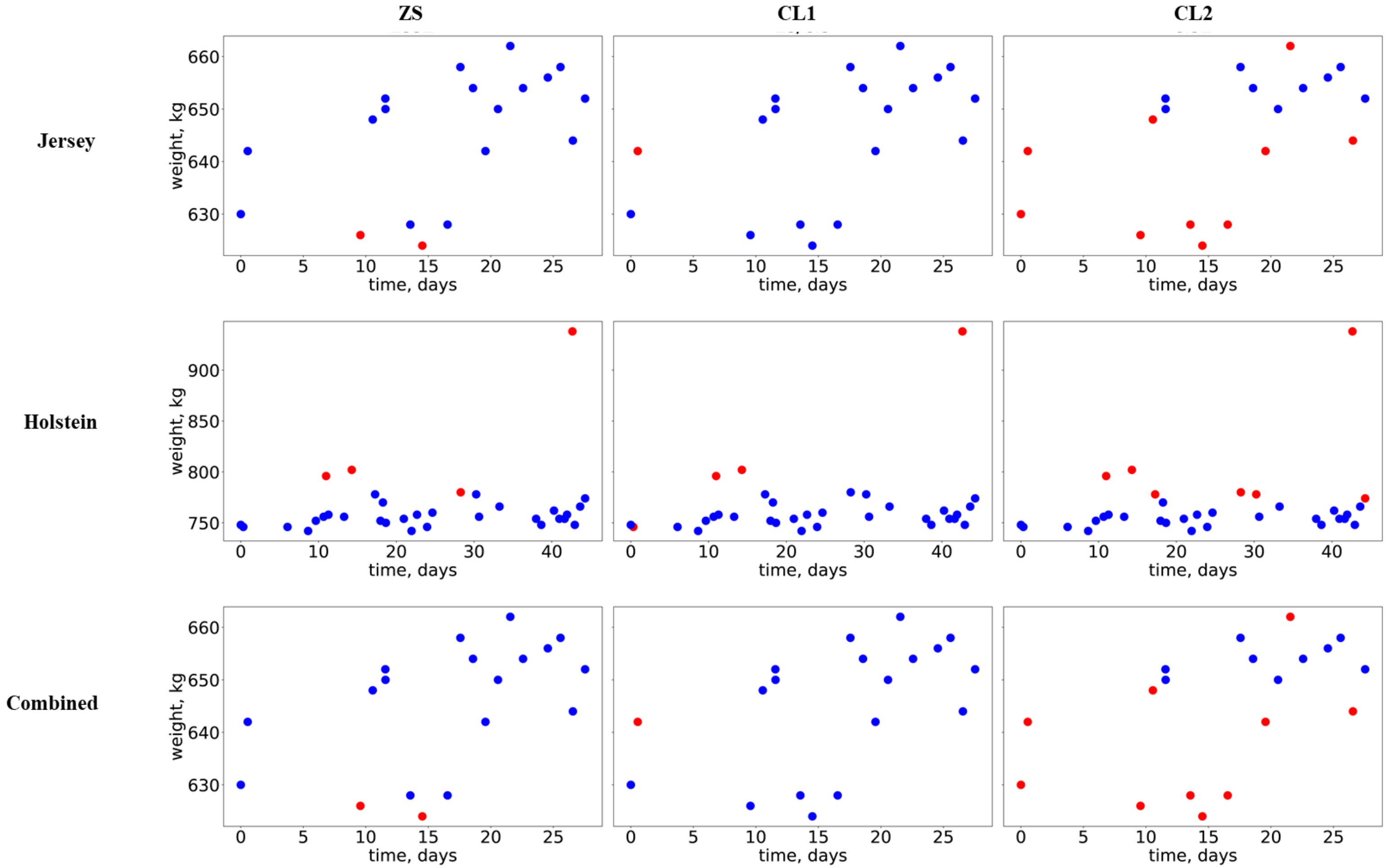

Fig. S1.3. An overview of efficiency/effect of different filtering methods for specific (randomly selected) individuals. Rows represent a specific caw from the relevant data source; columns represent different filtering (outlier removal) methods: ZS – modified Z-score; CL1 and CL2 – variants of clustering (see the main text for details); the numbers above each plot show amount of outliers detected; red dots – outliers, blue dots – filtered data.

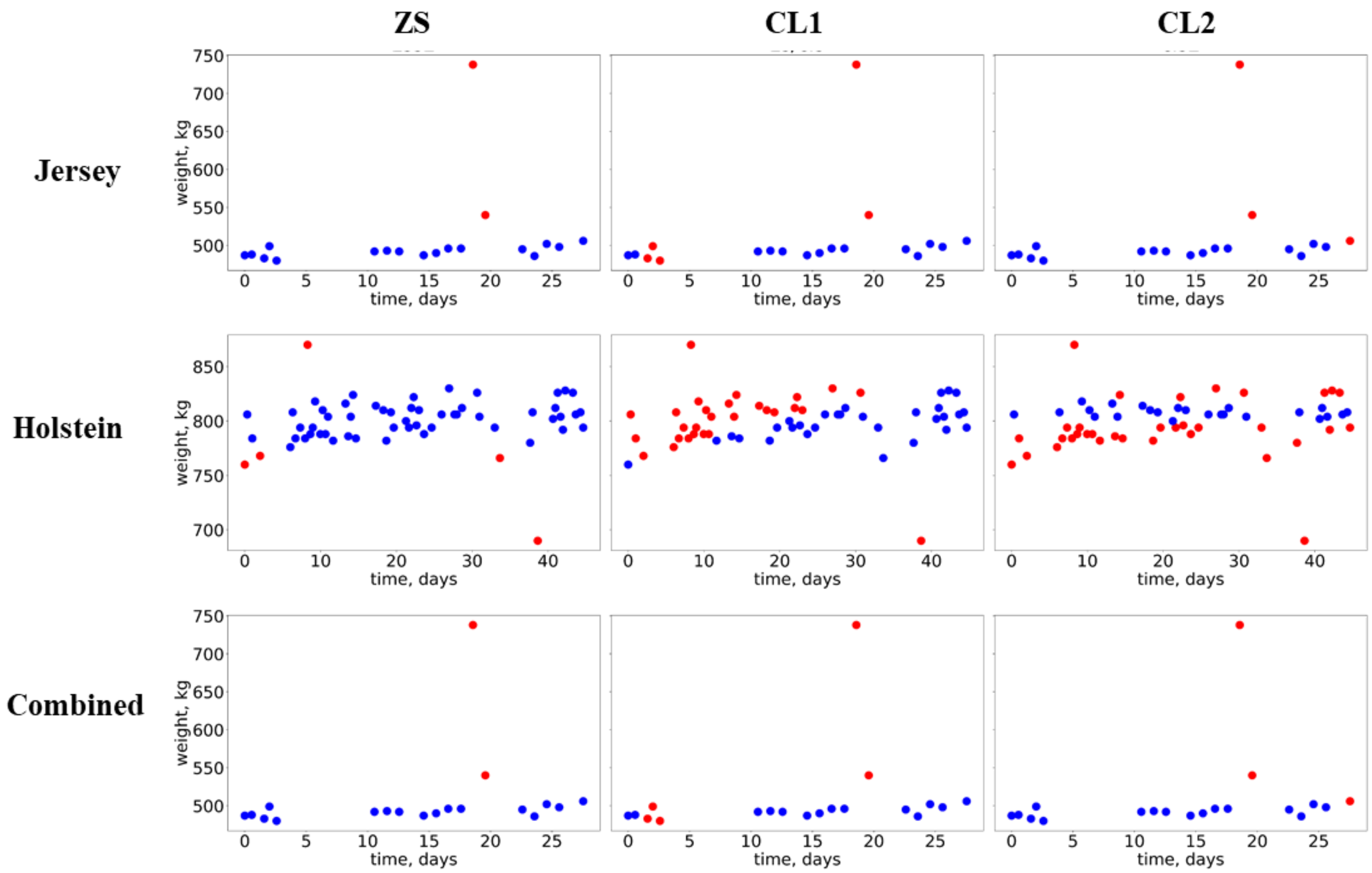

Fig. S1.4. An overview of efficiency/effect of different filtering methods for specific (randomly selected) individuals. Rows represent a specific cow from the relevant data source; columns represent different filtering (outlier removal) methods: ZS – modified Z-score; CL1 and CL2 – variants of clustering (see the main text for details); the numbers above each plot show amount of outliers detected; red dots – outliers, blue dots – filtered data.

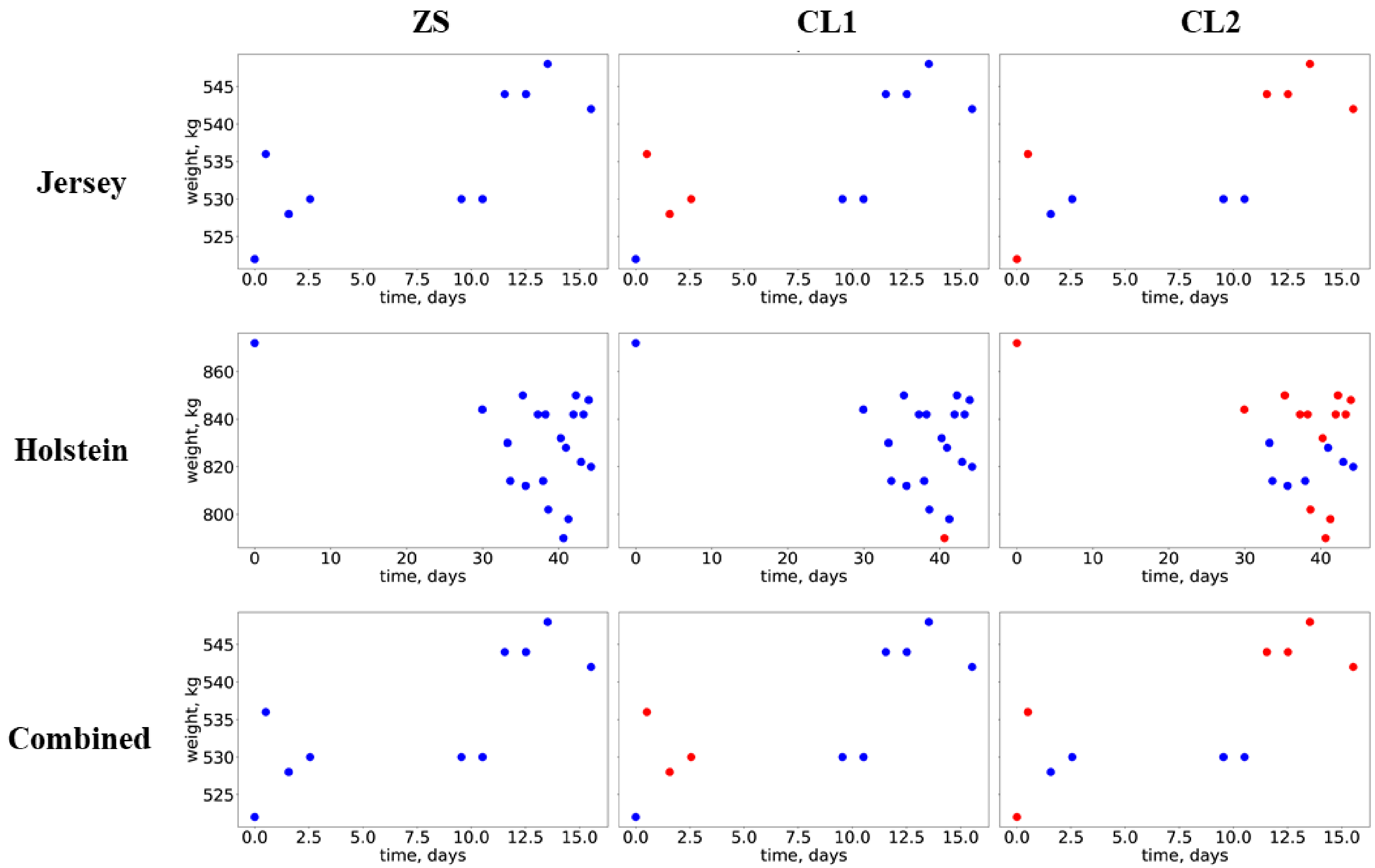

Fig. S1.5. An overview of contours imputation results for specific (randomly selected) individuals from different data sources. Rows represent a specific cow from the relevant data source; columns represent temporal contours changes; blue dots represent raw contour data, red do ts – imputed data.

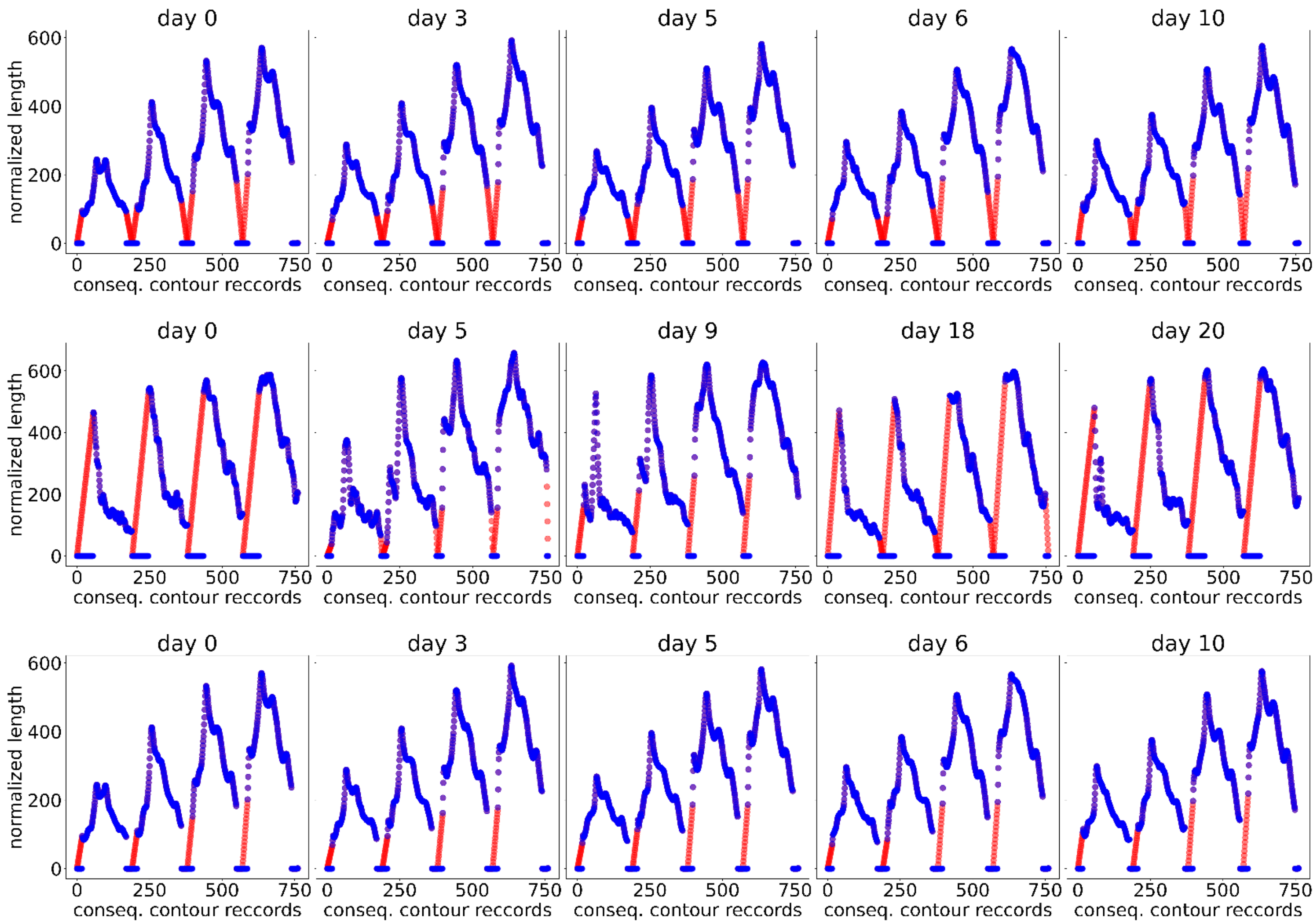

Supplement: Supplementary file 5 [file DataSheet1.PDF]
